# Supplementary material for: Are carbon emissions trading and green financial instruments synergistic? -Comprehensive quantitative research based on content analysis
Source: PLoS One. 2024 Mar 7;19(3):e0298601. doi: 10.1371/journal.pone.0298601 (PMC10919601; doi:10.1371/journal.pone.0298601)
Supplement: S1 Data — (ZIP) [file pone.0298601.s001.zip › Data/地级市Z_F工作报告.zip!/╣π╬≈/─╧─■╩╨/└┤╫╘╣1⁄2╓┌║┼í╛┬φ┐╦╩2╛▌═°í┐_2018.docx]

各位代表：

　　现在，我代表市人民政府向大会作政府工作报告，请予审议，并请各位政协委员和列席会议的同志提出意见。

　　一、2017年工作回顾

　　2017年，是南宁发展历程中极不平凡、砥砺奋进的一年。全市各级各部门认真学习贯彻党的十九大精神，坚持以习近平新时代中国特色社会主义思想为指导，贯彻落实习近平总书记视察广西重要讲话精神，不折不扣落实中央、自治区和市委的决策部署，牢固树立和践行新发展理念，真抓实干，攻坚克难，加快推进“六大升级”工程，经济发展稳中有进、稳中向好，社会保持和谐稳定，全面完成了市十四届人大二次会议确定的主要目标任务。

　　——经济发展实现新跨越。预计，全市地区生产总值、固定资产投资、全部工业总产值均突破4000亿元，分别增长8%、12%和14%；社会消费品零售总额突破2000亿元，增长11%；财政收入687.98亿元，增长11.95%；农林牧渔业总产值716亿元，增长4％，主要经济指标超额完成全年目标，好于预期，好于全国全区。

　　——“南宁渠道”影响力持续提升。圆满服务第14届中国—东盟博览会和商务与投资峰会，成功举办环广西公路自行车世界巡回赛（南宁站）、南宁国际马拉松比赛等重大赛事（活动），与东盟及其他“一带一路”沿线国家和地区的经贸、科技、文化等交流合作不断深化。预计，全市外贸进出口总值584.79亿元、增长40.5%，成为全国外贸百强城市；第二轮加工贸易倍增计划实现“开门红”，加工贸易进出口增长47%，总量占全区比重达49%。

　　——“绿城”品质魅力彰显。轨道交通2号线开通试运营，地铁进入换乘时代。基本完成国家海绵城市建设试点三年任务，那考河流域综合整治项目荣获“中国人居环境奖”范例奖。基本消除建成区黑臭水体。市区空气质量优良率为92.3%，其中优等级的天数创2013年实行环境空气质量新标准以来最高纪录，达191天，“南宁蓝”保持常态。实现全国文明城市“四连冠”。通过国家森林城市复查。跻身中国社科院发布的绿色发展和健康宜居城市全国前十名。在中国生态文明论坛（惠州）年会上获“2017美丽山水城市”称号。

　　——重点改革富有成效。在全国首创公共资产负债管理智能云平台，对政府公共资产负债的管控和监测进一步加强。市行政审批局规范运行，29个市直部门182项行政许可事项统一由市行政审批局行使，实现“一枚公章管审批”，我市“135审批体制改革路径”获“中国政务服务突出贡献奖”。不动产登记改革举措获国土资源部批示推广。

　　——法治南宁提上新水平。法治政府建设扎实推进，《中国法治政府评估报告（2017）》综合评价100个主要城市，我市名列第5位、西部城市第1位。

　　——民生福祉大幅提升。全年财政民生支出500.73亿元、增长14.97%，占一般公共预算支出的77.48%。预计8.37万贫困人口脱贫，101个贫困村摘帽。预计，全市居民人均可支配收入24883元、增长8.8%，居民消费价格指数上涨2.3%。城镇新增就业7.7万人，城镇登记失业率2.63%。

　　一年来，我们主要做好了以下工作：

　　（一）坚持调结构、聚动能，产业转型升级成效显著。

　　坚持“二产补短板、三产强优势、一产显特色”，以“稳”为基础，以“进”为目标，着力发展实体经济。预计，工业对经济增长的贡献率达34.5%、同比提高10.8个百分点，三次产业的比例从2016年的10.8︰38.5︰50.7优化为10︰38.9︰51.1。

　　创新动力持续增强。深入实施创新驱动发展战略，突出科技创新对供给侧结构性改革和培育发展新动能的支撑引领作用。预计科技创新对经济增长贡献率达57%。南宁·中关村创新示范基地累计引进行业重点企业33家、入孵创新团队52个、合作高校26所，初步形成智能制造等四个产业微集群，南宁·中关村创新示范基地产业园建设稳步推进。南宁高新区成为广西首个国家双创示范基地。高新技术产业不断壮大，高新技术企业达451家、占全区总量的三分之一，高技术产业产值预计完成760亿元、增长15%，南南铝跨入世界高端铝材加工行业。新建国际科技合作基地15家，南宁学院与科大讯飞合作建设广西首家人工智能学院。新增国家级科技企业孵化器2家、众创空间3个，建成自治区重点实验室4家。实施广西科技成果转化大行动项目76项，中国—以色列科技成果交流转化中心落户南宁。预计全市每万人口发明专利拥有量8.2件、达到全国中等水平。

　　加快补齐二产短板。深入实施“工业强市”战略，引导各类要素向工业经济聚集发力，工业新动能逐渐形成。预计，全市规模以上工业增加值完成1160亿元，增长10%；电子信息、先进装备制造、生物医药三大重点产业产值增长17%，占全市规模以上工业产值的比重达38.5%、同比提高约1个百分点；三大国家级开发区对全市工业增长贡献率达66%；亿元以上企业达660家、同比增加27家。富士康、源正等龙头企业产能持续释放，申龙客车、瑞声科技等一批项目落地，博世科环保产业园高安基地等23个重点工业产业项目建成投产。市本级工业用地成交面积增长108%，创近四年新高。建筑业加快发展，新增入库建筑企业36家。

　　服务业升级增效。深入实施金融业、现代物流业、电子商务和信息服务业“三年行动计划”，千方百计优供给促消费。预计第三产业增加值突破2100亿元，增长8.5%。获认定自治区级现代服务业集聚区9家。坚持实施“引金入邕”战略，金融服务实体经济发展能力增强，新增新三板挂牌企业11家，中国进出口银行广西区分行等金融机构开业，村镇银行实现县域全覆盖，预计金融业增加值达450亿元、对经济增长贡献率为10.8%。发展壮大现代物流业，新增3A级以上物流企业5家、总数达17家；预计，快递业务量增长35%，公路、水路客货运周转量分别增长9%、20.5%。加快发展电子商务和信息服务业，五象新区总部基地休闲公园电商小镇建成运营，全国首创建成集国际邮件、跨境电商、国际快件监管于一体的中国邮政东盟跨境电子商务监管中心，预计全市重点企业电子商务交易额2500亿元、增长13.6%，软件和信息技术服务业主营收入130亿元、增长8.3%。举办超万平方米展会45场。万达茂等大型城市综合体开业运营。入围国家中医药健康旅游示范区创建单位，接待旅游总人数突破1亿人次，旅游总消费突破1000亿元。

　　现代特色农业提质发展。深入推进农业供给侧结构性改革，着力增加绿色优质农产品供给。预计全市完成粮食总产量217万吨，宾阳县获“中国好粮油”行动示范县。糖料蔗恢复性增长，“双高”基地达17.3万亩。全市在建各类特色农业基地112个，其中高标准“菜篮子”基地65个。我市成为全国火龙果和沃柑最大生产地。5个农产品入选全国名特优新农产品目录，新增“三品一标”农产品17个、国家地理标志保护产品1个、国家地理标志商标2个、富硒农产品认证15个，横县茉莉花茶被评为“中国优秀茶叶区域公用品牌”，上林县成功创建国家有机产品认证示范区。新增广西现代特色农业（核心）示范区8个，西乡塘“美丽南方”休闲农业（核心）示范区入选国家农业综合开发田园综合体建设试点项目。大力推进乡村旅游连点成线提质增效，新增广西休闲农业与乡村旅游示范点7个，马山县获评全国休闲农业和乡村旅游示范县。

　　（二）坚持优服务、推项目，经济发展后劲明显增强。

　　坚持“一线”工作法，强化土地、资金等要素保障，狠抓重大项目建设，发挥投资对稳增长的积极作用。

　　重大项目顺利推进。按照投资有效益、企业有利润的要求，实行投资项目目录清单管理，加快现代产业、社会民生、基础设施等重点领域重大项目建设。预计亿元以上项目完成投资1181亿元、增长18%。553项区市层面统筹推进重大项目预计完成投资960亿元，拉动投资增长8.5个百分点，南宁牛湾港疏港大道等74个项目竣工。城建计划项目加快推进，预计完成投资476亿元。重大公益性项目扎实推进，广西文化艺术中心、青山大桥、南宁国际会展中心改扩建工程场馆等建成使用。

　　投资结构持续优化。狠抓工业投资和民间投资两个关键，预计，工业投资完成1075亿元、增长7%，其中电子信息、先进装备制造、生物医药三大重点产业完成投资438亿元、占工业投资的40%；民间投资完成2801亿元、增长14%，占全市投资的64%。基础设施投资、社会事业投资预计分别完成1058亿元、273亿元，增长20%、25%。积极拓宽融资渠道，新落地PPP项目9个，涉及总投资116.23亿元。

　　营商环境更加便利。做好简政放权的“减法”，取消、调整334项行政审批事项，推行“容缺后补”审批制度；南宁市民中心启用，60个政府职能部门和公共企事业单位共432项政务服务事项集中入驻；137项政务服务事项可通过网上审批大厅办理，实现“零见面、零跑腿”。做好优化服务的“乘法”，在全区率先开展个体工商户简易注销改革，全面落实“39证合一”登记制度、企业简易注销、商标注册便利化等改革；设立中小微企业孵化基金，充分发挥“两台一会”中小企业贷款平台作用，有效缓解中小企业融资难问题。做好加强监管的“加法”，扎实推进“双随机一公开”监管改革，搭建公共资源交易市县一体化“1+6”新平台，交易大数据系统在全区率先上线运行。市场经营环境不断改善，市场活力不断激发，新增市场主体10.98万户。

　　（三）坚持建平台、扩贸易，“南宁渠道”进一步畅通。

　　积极融入“一带一路”建设，突出平台支撑、交通支撑、产业支撑，推进“南宁渠道”升级，开放合作水平迈上新台阶。

　　开放平台支撑有力。积极融入中新互联互通南向通道建设，中新南宁国际物流园启动建设，中欧班列（南宁—河内）集装箱班列开通。中国—东盟信息港南宁核心基地建设扎实推进，广西电子政务外网云计算中心等10个项目竣工。南宁综合保税区封关运营，入驻企业34家。第十二届中国（南宁）国际园林博览会成功邀请19个东盟及“一带一路”沿线国家城市参展。香港特区政府驻广西联络处在邕揭牌成立。

　　互联互通基础夯实。加快完善公路、铁路、民航、水运立体综合交通网络，贵隆高速公路等项目加快推进，南宁—贵阳高铁、南宁—崇左城际铁路开工建设，南昆铁路南宁至百色段增建二线、黎湛铁路电气化改造项目建成通车。吴圩国际机场旅客年吞吐量达1391.2万人次、增长20.3%。老口航运枢纽发挥效益，邕宁水利枢纽船闸工程蓄水通航。

　　开放格局持续扩大。大力发展开放型经济，新增进出口实绩企业181家，引进加工贸易企业12家，培育形成富桂精密等一批出口品牌标杆。坚持以上率下招商，成功签约华润基金、宜家家居等416个项目。CEPA先行先试取得新成效，香港农本方等项目落地。预计，全市实际到位资金突破800亿元、增长14%，其中全口径实际利用外资9.4亿美元、增长22.1%。

　　（四）坚持提品质、优环境，生态宜居水平明显提升。

　　坚持“治水、建城、为民”的城市工作主线，厚植绿色发展优势，保持南宁青山常在、清水长流、空气常新。

　　五象新区发展再提速。新区全年在建项目547个、新开工项目136个（含子项目）、竣工项目54个，固定资产投资完成355亿元、增长17.6%。加速产城融合布局，引进广西—东盟地理信息与卫星应用产业园等一批项目，建成南宁现代化建材加工及物流配送中心一期工程等一批项目。加快完善新区基础设施，3座立交桥、28条道路建成通车，市国家档案馆（方志馆）等一批公益性项目竣工，十四中五象校区等一批中小学校招生，给排水、电力、燃气等配套项目同步建成。扎实推进国家级绿色生态示范区创建，新区核心区绿色建筑比例达100%。

　　打造优美生态环境。持续深化大气污染治理，PM10、PM2.5浓度分别较上年下降9.7%、2.8%，市区空气质量综合指数在全国各省会城市（含直辖市）中排名第6，“南宁蓝”成为市民生态之福。全面推行河长制，建立覆盖市县乡村四级河长组织体系，打好治水“组合拳”。总结那考河整治的成功做法，形成流域治理可复制可推广的南宁经验，南宁水“清”出新典范。全面实施邕江两岸148公里岸线综合整治，建成两岸31公里景观带，邕江上游引水工程开工。沙江河、心圩江、水塘江等城市内河整治提速。全国水生态文明城市建设试点通过验收。深入推进城市绿化美化彩化，新建自然保护小区17个、绿道34公里。按国家5A级旅游景区标准全面铺开园博园建设，园区面貌勾勒成型。打造环广西公路自行车世界巡回赛（南宁站）沿线景观带，马山“最美赛道”广受好评。推进速生桉林地林种结构调整，改善水源地森林生态环境。横县西津国家级湿地公园试点建设通过验收。万元GDP能耗预计下降4%以上。全面完成中央环保督察反馈意见整改。

　　有序推进城市建设。加快新一轮城市总体规划修编，形成南宁城市总体规划（2017—2035年）基本战略框架。启动总体城市设计，入选全国第二批城市设计试点城市。积极创建公交都市，地铁、快速公交日均客运量分别达26.6万、13.5万人次，市区内公交500米覆盖率达99.5%。拓展加密城市路网，中华—园湖立交、城市东西向快速路东段等重要交通节点项目建成，开展嘉园小区等4个片区街区制改造，打通道路微循环，五一路铁路桥段等一批“瓶颈路”“断头路”拓宽或打通。全面开展“城市双修”，不断提升受污染耕地、污染地块安全利用率；完成年度棚户区改造任务，开工建设“三街两巷”核心区（一期），实施老旧居住区综合整治改造100个。新建成地下综合管廊26.13公里。新建装配式建筑达62万平方米。以BIM等信息技术推动工程质量提升，3个项目同时获中国建筑工程鲁班奖。

　　科学有效治理城市。城市综合治理常态化，扩大城管综合行政执法范围，实现住房城乡建设领域行政处罚、行政强制权的集中行使。加快智慧城市建设，建成全国首个海绵防涝信息系统——南宁市市区防涝预警监控信息系统。深入开展“美丽南宁·整洁畅通有序大行动”，推进“智慧交通”建设，规范网约车、电动自行车、共享单车等管理，形成城市道路交通管理的南宁经验，“文明行车·礼让斑马线”主题活动获中央媒体点赞。

　　（五）坚持强基础、兴产业，县域经济发展迈上新台阶。

　　把促进县域经济发展放在更加突出的位置，以城乡一体、产业带动、基础支撑为突破口，推动县域经济稳步发展。

　　新型城镇化步伐加快。强化规划管理，全市各级土地利用总体规划调整成果获批实施。大力实施大县城战略，加快以县城为中心的重点镇建设，横县校椅镇入选第二批全国特色小镇、六景镇全面启动第三批国家新型城镇化综合试点工作，宾阳县自治区新型城镇化示范县工程持续推进，完成马山县古零镇、西乡塘区金陵镇自治区百镇建设示范工程。深化户籍制度改革，从教育、医疗、社保、就业、住房等方面加快农业转移人口市民化进程。

　　县域经济实力不断增强。落实主体功能区规划，因地制宜打造“一县一拳头产业”。横县现代农业产业园入选创建第一批国家现代农业产业园，宾阳县入选国家农业综合开发现代农业园，上林县获国家生态文明建设示范县，中国首个攀岩特色体育小镇落户马山县，隆安县金穗生态园获全国休闲农业与乡村旅游示范区“五星级”称号。江南区、青秀区、邕宁区、武鸣区获评2016年度广西科学发展进步县（区）。兴宁区获评2017年度全国投资潜力百强区，青秀区连续两年获评全国“四个百强区”，良庆区被认定为第二批广西可持续发展试验区创建单位。

　　县域综合承载能力提升。建立健全县域基础设施、公共服务设施能力提升“三年行动计划”项目库，分级分批推进1288个项目实施。以开展“美丽南宁·宜居乡村”活动为抓手，增强服务功能，夯实基础支撑，1383个行政村建成并挂牌村级综合服务中心，硬化350公里非贫困村通屯道路，建成113个行政村光纤网络工程试点项目，新、改建输配电线路1050公里，22个镇级污水处理设施项目通水试运行，完成改厨改厕改圈年度目标任务，农村人居环境明显改善。

　　（六）坚持抓改革、破难点，发展活力动力充分释放。

　　坚持问题导向，聚焦重点领域和关键环节，坚定不移推进全面深化改革。

　　“三去一降一补”任务有效落实。化解过剩水泥产能60万吨，全面完成“地条钢”取缔任务，完成77家国有“僵尸企业”职工分流安置。从土地供应、信贷政策等方面加强房地产市场监管，保持市场供需平稳。建立健全政府性债务风险应急处置工作机制，切实防范和化解财政金融风险。全面落实自治区、市降成本各项政策措施，预计全年为企业减负超42亿元。民生短板加快补齐，预计教育领域、卫生和社会工作领域投资分别增长26.9%、49.8%。

　　重点领域改革统筹推进。承担的26项国家级和28项自治区级改革试点进展顺利，市本级156项年度改革任务基本完成。国资国企改革力度加大，13家企业混合所有制试点稳妥推开。财政改革步伐加快，中期财政规划编制实现市本级预算单位全覆盖，纳入政府购买服务预算管理项目455项、增长19.1%。沿边金融综合改革深入推进，16家驻邕银行机构接入人民币跨境支付系统，跨境人民币结算量超150亿元。持续推进“农金村办”模式，全市“三农金融服务室”覆盖面达69%，武鸣区农村承包土地经营权抵押贷款试点工作成效明显。深化农村综合改革，农村土地承包经营权可颁证率达96.1%。国有林场主体改革基本完成。土地二级市场试点改革有序推进，探索搭建二级市场交易平台。

　　（七）坚持惠民生、增福祉，群众获得感不断增强。

　　坚持集中力量做好普惠性、基础性、兜底性民生建设，加快推动民生福祉升级，让更多改革发展成果与民共享。

　　年度脱贫攻坚任务顺利完成。深入推进“七个一批”“七大工程”，筹集安排各级财政专项扶贫资金23.33亿元投入脱贫攻坚。培育和引导149家龙头企业、1239个农民合作社积极参与产业扶贫，带动23万多名贫困人口增收。年度易地扶贫搬迁安置点全部开工建设，累计搬迁入住18258人。317个贫困村村级集体经济收入均达2万元以上。集中研究破解深度贫困问题，落实61家企业结对帮扶56个深度贫困村，由华润集团对口帮扶隆安县都结乡。扎实推进茂名-南宁扶贫协作。

　　各项社会事业持续发展。教育事业加快发展。着力解决幼儿“入园难”，建成18所公办幼儿园。义务教育均衡发展取得新成效，4个县区通过国家义务教育均衡发展督导评估认定，学区制管理改革加快推进，集团化办学模式不断拓展，建成并投入使用公办中小学校18所。接收进城务工人员随迁子女入学约14.5万人。基本普及高中阶段教育，2017年高考成绩领跑全区。中高职教育与重点产业融合发展取得新成效，南宁职业技术学院入选国家第二批现代学徒制试点院校。南宁教育园区累计签约入驻院校16所，其中开工建设6所、实现办学招生1所。千方百计促进创业就业。扶持创业1.92万户，发放创业担保贷款3220笔共2.74亿元；为高校毕业生等各类群体提供就业岗位超26万个。健康南宁建设扎实推进。在全区率先启动实施统一的城乡居民医保制度，实现医疗费用即时结算。在全区首创职工医保“家庭共享”；推进智慧健康信息工程（一期），初步实现13家市直属医院化验、检查、档案查阅结果的互认共享。城市公立医院综合改革稳步推进，实现药品“零差价”。上林县医改模式上升为国家推广的医疗共同体改革模式。全面两孩政策顺利实施，荣获“全国创建幸福家庭活动示范市”称号。社会保障水平稳步提升。城乡居民基本养老保险和基本医疗保险参保率分别达95%、98.95%。企业退休人员基本养老金实现“十三连涨”。城市居民最低生活保障标准提高20%。新增医养结合机构12家，新、改、扩建社区日间照料中心30个。基本建成公共租赁住房2.73万套，分配入住2.39万套。文化和体育事业繁荣发展。持续打造民歌湖大舞台周周演群众性文化活动等特色文化品牌。推动媒体融合发展，精心推出80多个主题宣传专栏，举办中国—东盟（南宁）戏剧周等60多项大型主题文化活动。深化中国—东盟（南宁）文化、媒体交流合作机制，与50多家境内外文化传媒机构达成实质性合作。大型方言话剧《水街》等4部作品入选广西第14届精神文明建设“五个一工程”。广泛开展全民健身活动，举办市、县级全民健身赛事活动500多项，成功申办2019年苏迪曼杯世界羽毛球混合团体锦标赛。

　　推动社会治理创新。深入推进平安南宁建设，积极构建“七位一体”立体化社会治安防控体系，牢牢守住“不打响、不炸响”的反恐维稳工作底线，“智慧警务”积极推进，严厉打击网上虚假信息诈骗等违法犯罪行为，应急管理机制不断完善，人民群众安全感满意度进一步提升。落实市四家班子领导包案制，突出矛盾纠纷问题有效化解。隆安县获评全国平安建设先进县。村（社区）“两委”换届选举工作全面完成。守信联合激励和失信联合惩戒大格局加快构建。健全落实安全生产“党政同责、一岗双责、齐抓共管、失职追责”责任体系，全年无重特大生产安全事故发生。全面实施食品安全战略，落实“四个最严”“四有两责”要求，食品药品安全形势总体稳定，横县获评“国家级出口食品农产品质量安全示范区”。

　　一批惠民实事顺利实施。基本完成自治区级和市本级为民办实事工程共71个子项。

　　（八）坚持提效能、转作风，政府自身建设呈现新气象。

　　坚持依法行政、从严治政、廉洁勤政，政府自身建设不断加强。法治政府建设加快。提请市人大常委会审议地方性法规草案4件，出台政府规章3件、规范性文件40件，组织清理政府规章和规范性文件近5000件，废止规章4件、修改2件，宣布失效文件3700余件、决定修改13件。公共法律服务体系加快建设， “七五”普法深入推进。科学决策水平不断提升。认真执行市人大及其常委会的决议、决定，依法接受市人大及其常委会的法律监督和工作监督，主动接受人民政协的民主监督和社会舆论监督，共办理自治区和市级人大代表议案建议220件、政协提案388件，办结率100%。进一步密切与各民主党派、工商联、无党派人士、人民团体和社会各界人士的联系。落实重大决策社会稳定风险评估机制，完成“南宁市主体功能区规划”等重大行政决策后评估。加强政府智库建设，聘任政府参事6名、第二届专家咨询委员会知名专家55名。作风建设持续加强。严守党纪党规，严格落实中央八项规定精神和国务院“约法三章”，深入推进“两学一做”学习教育常态化制度化，驰而不息纠正“四风”，“三公”经费同比下降12.12%。政务公开透明度在全国49个较大城市中位列第10。我市连续7年被评为人民网网友留言办理工作先进单位。举办电视问政节目10期、53个问题完成整改。强化对扶贫等重点领域、关键岗位的审计和监督执纪问责，严查发生在群众身边的腐败问题，促进政府系统廉洁行政。

　　支持国防和军队改革，巩固军政军民团结。此外，保密、台湾事务、档案、民族宗教、统计、侨务、人防、地震、机关事务管理、地方志、水库移民、供销、双拥、出版、消防、海关、海事、检验检疫、税务、调查、测绘、气象、水文、文史、哲学和社会科学等工作扎实推进，妇女、儿童、老年人、残疾人等事业健康发展。

　　各位代表，过去一年的成绩来之不易。这是中央、自治区和市委正确领导的结果，也是全市各族人民同心同德、顽强拼搏的结果。在此，我代表市人民政府，向全市各族人民，向人大代表、政协委员，向各民主党派、工商联、无党派人士、各人民团体和社会各界人士，向驻邕人民解放军、武警部队官兵，向所有关心支持南宁建设发展的港澳台同胞、海外侨胞及国际友人，表示衷心的感谢并致以崇高的敬意！

　　各位代表，在看到成绩的同时，我们也清醒地认识到发展中的困难和问题：经济发展稳的基础还不够牢固，进的动能还不够强劲，补齐民生短板和脱贫攻坚任务还比较艰巨，政府系统少数干部担当意识还不够强，等等。我们将正视问题，切实采取措施加以解决。

　　二、2018年工作安排

　　2018年是贯彻党的十九大精神的开局之年，是改革开放40周年，是决胜全面建成小康社会、实施“十三五”规划承上启下的关键一年，是自治区成立60周年，做好今年各项工作，具有特殊的意义。我们必须凝心聚力、砥砺奋进，勇于担当、善做善成，奋力推动首府经济社会向更高质量发展。

　　今年政府工作的总体要求是：全面贯彻党的十九大精神，以习近平新时代中国特色社会主义思想为指导，深入贯彻落实中央、自治区经济工作会议以及市委十二届四次、五次全会的决策部署，坚持稳中求进工作总基调，坚持新发展理念，紧扣我国社会主要矛盾变化，按照高质量发展的要求，统筹推进“五位一体”总体布局和协调推进“四个全面”战略布局，坚持以供给侧结构性改革为主线，统筹推进稳增长、促改革、调结构、惠民生、防风险各项工作，大力推进改革开放，落实国家宏观调控政策，推动质量变革、效率变革、动力变革，坚决打好防范化解重大风险、精准脱贫、污染防治三大攻坚战，持续推进“六大升级”工程，促进经济社会持续健康发展，为加快建设“四个城市”，勇当广西持续营造“三大生态”、加快实现“两个建成”的排头兵，奋力谱写新时代南宁发展新篇章奠定坚实基础。

　　今年全市发展的主要预期目标是：地区生产总值增长7.5%，财政收入增长7%，固定资产投资增长10.5%，规模以上工业增加值增长8.5%，社会消费品零售总额增长10%，外贸进出口总值增长10%，全市居民人均可支配收入增长9%，居民消费价格指数上涨幅度控制在3.5%以内，城镇登记失业率控制在4%以内，城镇新增就业6.5万人，节能减排降碳控制在自治区下达目标内。

　　围绕上述目标，我们要着力做好以下八个方面工作：

　　（一）做大做强实体经济，持续推进产业转型升级。

　　坚持质量第一、效益优先，持续按照“二产补短板、三产强优势、一产显特色”的思路，以供给侧结构性改革为主线，把提高供给体系质量作为主攻方向，促进经济高质量发展。

　　推动工业提质发展。坚持“工业强市”战略不动摇，以三大重点产业为着力点、工业园区为依托、重点企业为龙头、项目建设为抓手，拉长拓宽延伸产业链，加快打造千亿元产业集群。推动制造业的“进”。发展实体经济，重点在制造业、难点也在制造业。立足我市工业发展实际，集中发力以电子信息、先进装备制造、生物医药为代表的先进制造业，不断提高三大重点产业产值占全市规模以上工业产值的比重。电子信息产业方面。打造以富士康、瑞声科技为龙头的电子信息产业集群，继续在高新区、江南工业园等园区大力引进上下游产业和相关配套产业。在经开区建设新一代信息技术产业发展载体，培育发展微电子产业。大力发展中高端电子信息产品制造，推动大数据、云计算、物联网、人工智能等智慧产业集聚发展，加快广西北斗综合位置服务平台建设，争取南宁北斗信息产业园等项目投入运营。力争电子信息产业产值突破600亿元。先进装备制造产业方面。推动申龙客车、白马环卫车、同捷新能源汽车等项目开工建设，扶持玉柴专汽扩大新能源物流车规模，打造以源正为龙头的新能源汽车产业集群。推进南南电子汽车新材料精深加工等项目建设，发展航空、轨道交通、汽车、IT等领域的高端铝材，打造以南南铝为龙头的新材料产业集群。支持中车精密、中铁广发公司发展壮大，打造以中车为龙头的轨道交通装备产业集群。力争先进装备制造产业产值突破900亿元。生物医药产业方面。着力引进一批国内外知名生物医药及相关配套产业企业，加快广西医疗器械检测中心及培力药业异地扩能等项目建设，重点推进葫芦娃、医大仙晟、鸿博原生药业等一批项目通过生产行政许可并试产，打造以柳药、海王、培力等为重点的生物医药产业集群。力争生物医药产业完成产值180亿元。推动传统产业的“转”。传统产业是我市工业的家底和支柱。实施传统优势产业“二次创业”，研究出台振兴传统产业的政策措施，鼓励引入新技术、新管理、新模式，鼓励使用地方名特优产品，促进越来越多的传统产业在转型升级中“凤凰涅槃”。支持化工、建材、造纸和木材加工等企业改造提升，推进食品、轻纺等消费品工业增品种、提品质、创品牌，力争食品加工产业产值率先突破1000亿元。实施绿色制造工程，深入推进高新区国家低碳试点园区建设，加快经开区、东盟经开区循环化改造。加快对新兴产业的“育”。积极培育发展以高端铝材、石墨烯等为重点的战略性新兴产业，加快南宁科天水性科技产业园和牛湾港、六景、伶俐装配式建筑产业基地等项目建设，培育工业发展新动能。加大对工业经济的“扶”。着力解决国家级开发区空间拓展问题，以飞地园区的方式与县区工业园区开展合作共建，力争高新区、经开区规模以上工业增加值增长15%以上，东盟经开区规模以上工业增加值增长18%以上。加大工业用地供给，全面开展工业闲置用地清理工作，提高工业用地效益。发挥人力资本作用，注重激发和保护企业家精神，培育富于创新和敢闯敢为的企业家；大力弘扬劳模精神和工匠精神，继续推进农民工职业技能提升培训计划，加快建设知识型、技能型、创新型劳动者大军。

　　提升服务业发展水平。以加快现代服务业集聚区建设为抓手，建立服务业发展分类考核机制，发展壮大三大重点服务业产业，促进服务与制造协同发展。推动金融业持续健康发展。大力实施“引金入邕”“引资入邕”，积极推动在CEPA项下组建合资证券公司，推动渤海银行等金融机构在邕设立分支机构，推进平安银行南宁分行、国富人寿保险公司开业运营，力争中银香港在邕设立东盟后台服务中心。加强政金企沟通合作，引导金融加大服务实体经济力度。积极发挥财政金融的协同作用，争取更多金融资源倾斜。加大政策性保险力度，持续推进政策性农业保险和科技保险试点。加快发展现代物流业。优化物流通道，发展“公铁”“水铁”“陆空”等多式联运；加快建设物流集聚区，重点抓好中新南宁国际物流园、中国—东盟国际物流基地、空港物流园、牛湾物流园等建设；鼓励大型（零售）企业自建现代物流，加快推进传统物流业由单纯仓储货运向全物流产业链升级，加快建设连接东盟的重要物流节点城市。大力发展电子商务和信息服务业。深入推进“电商南宁”工程，实施电子商务提升工程，引导传统商贸企业转型升级，培育壮大本地电子商务龙头企业，加快高新区国家电子商务示范基地、中国—东盟（南宁）跨境电子商务产业园等项目建设，完善和拓展跨境电子商务业务；大力发展农村电商，推进宾阳县、横县、上林县全国电商进农村试点县工作，加快建设辐射东盟的电子商务中心城市。推进研祥智谷（二、三期）建设，加快供应链集成服务基地等建设，发展壮大信息服务业。大力发展旅游业。持续推进国家全域旅游示范区及国家中医药健康旅游示范区创建，重点推进百里秀美邕江、环首府生态旅游圈、环大明山生态旅游圈、南宁东盟文化博览园等项目建设，开展南宁市国际文化旅游休闲集聚区等项目前期工作。加快建设区域性国际消费中心城市。积极培育消费热点，大力发展品质消费、服务消费、绿色消费，加快发展轨道交通经济，增强消费对经济发展的基础性作用。统筹推进科技研发、咨询设计等现代服务业，支持发展分享经济、数字经济、创意经济等，持续推进青秀区楼宇经济示范区建设。

　　大力发展现代特色农业。把增加绿色优质农产品供给放在突出位置。严格落实粮食安全行政首长责任制，确保粮食总产量稳定在214万吨以上。严格落实耕地保护和占补平衡政策，推进农村土地综合整治，提高耕地质量。深入实施现代特色农业产业“10+3”提升行动，建设80个标准化、规模化种养殖基地，扩大富硒农业产业规模，延伸茉莉花产业链，做优香蕉、火龙果、晚熟杂交柑橘、优质大米等优势产业，推进生猪、渔业转型升级，加快肉牛羊产业发展，大力发展林下经济，培育一批农产品加工特色小镇，打造一批“邕系”农产品知名品牌。强化农业科技服务，推进有机肥替代化肥，开展优势水果病虫害统防统治，推进马山全国气象为农服务示范县建设。深化“产村互动、农旅融合”建设模式，务实推进现代特色农业示范区建设增点扩面提质升级，补齐示范区在种业、深加工、冷链物流和销售平台建设等方面的短板，升级打造一批县、市、自治区、国家级示范区。积极开展“三区三园一体”创建，争创更多的国家级、自治区级产业示范园区，重点加快横县国家现代农业产业园和全国糖料蔗生产保护区建设，加快推进西乡塘“美丽南方”国家农业综合开发田园综合体建设试点。持续推进休闲农业与乡村旅游发展，全力加快30个休闲农业示范区建设。

　　（二）坚持创新驱动发展，培育经济新动能。

　　坚持把创新作为第一动力，着力建平台、育主体、优生态，让适宜的种子在适宜的环境中开花结果。

　　加快建设创新平台。持续深化南宁·中关村创新示范基地建设，继续培育和引进一批科技领军企业，加快建设南宁·中关村创新示范基地产业园，进一步发挥基地示范引领和辐射带动作用。创建国家自主创新示范区，完善创业孵化链条，新建创业孵化载体4个以上，新增入孵企业和团队50个。加快推进高层次产学研合作，与高校院所共建院士工作站、科技企业孵化器等创新创业平台8个以上。加强国际科技合作基地建设，支持企业与越南、柬埔寨等东盟国家共建研发平台和技术转移中心。培育和提升南南铝加工研究院、富士康东盟硅谷科技园等创新平台，加快高新区生物工程技术中心等公共技术服务平台建设，提升工程技术研究中心20家、国际技术转移中心4个，力争新增市级以上企业技术中心、研发中心8家。

　　大力培育创新主体。鼓励技术创新和管理创新、商业模式创新融合，推动企业成为研究开发投入、技术创新活动和创新成果应用的主体。实施高新技术企业倍增计划和“瞪羚企业”培育计划，力争新增高新技术企业70家。推动科技型中小企业创新，通过科技创新券、科技信贷风险资金池、科技保险等扶持政策，支持科技型中小企业自主研发或技术引进。深入实施发明专利双倍增计划，争创国家知识产权示范城市，健全打击侵犯知识产权长效机制，强化知识产权创造、保护、运用。

　　持续优化创新生态。加快提高全社会研究与试验发展经费支出占全市地区生产总值的比重。深化科技体制改革，探索开展第三方专业机构管理科技项目试点。出台提升自主创新能力若干政策，加大对创新平台、人才和创业孵化的支持力度。实施重大科技专项10项、广西科技成果转化大行动项目76项。创建和提升6个自治区级农业科技示范园和10个农业科技扶贫示范基地。

　　（三）发挥投资对优化供给侧结构的关键性作用，积极扩大有效投资。

　　继续保持适当的投资强度，紧盯重点领域重大项目建设不放松，不断增强发展后劲。

　　促进有效投资特别是民间投资合理增长。积极谋划、储备、推进一批重大项目，完善领导联系重大项目、服务重点企业制度，建立健全市直部门服务、支持园区重大项目、重点企业工作机制，按照“四定”要求，全力推进区市层面统筹推进重大项目建设，争取完成投资750亿元以上。推动产业项目攻坚，重点推进农业、工业、服务业、建筑业投资额排名前30位的重大项目。推动民生项目攻坚，保持教育、卫生、扶贫、生态环保等社会民生领域投资增长不减。推动基础设施项目攻坚，加大城建计划项目投资力度，加快完善工业园区基础设施，着力抓好连接园区、县区的重点道路建设。激活民间投资，实施投资工程包，通过“捆绑打包”等方式加强政府和社会资本合作，强化项目策划包装和前期工作，推动PPP项目落地并规范实施。

　　全力推进重大公益性项目建设。按照“保进度、保质量、保安全”的要求，强化精品意识，全力服务好自治区统筹推进的迎接自治区成立60周年公益性项目建设，早日建成南宁国际会展中心改扩建工程、广西国际壮医医院等项目；如期竣工凤岭综合客运枢纽、城市东西向快速路、沙江河环境综合整治、南宁图书馆（新馆）、市儿童医院、南宁国际旅游中心（一期）等项目，“三街两巷”核心区（一期）开街运营，着力打造一批具有广西特色和时代风貌、获得广泛认可的民心工程，让首府南宁以崭新的城市面貌迎接自治区60周年大庆。

　　狠抓重大交通基础设施项目建设。积极配合建设吴圩经大塘至浦北、贵港经隆安至硕龙、柳州经合山至南宁等高速公路，加快张村至六景、新江至扶绥等公路项目建设，力争开工吴圩国际机场至隆安高速公路，加快建设南宁-贵阳高铁，推进南宁-崇左城际铁路建设，加快南宁-玉林城际铁路前期工作。推进吴圩国际机场军民分离，力争开工建设第二跑道，全面启动建设南宁国际空港综合交通枢纽（GTC），加快伶俐通用机场建设。推进牛湾、六景港口和码头建设，加快建设西津二线船闸，建成邕宁水利枢纽主体工程，提升西江航线的通航能力和港口集散功能。

　　拓宽投融资渠道。突出企业投资主体地位，加快建立投资项目管理负面清单、权力清单、责任清单，缩减企业投资项目核准范围，推进高新区开展企业投资项目承诺制试点工作。引入市场机制，积极探索融资租赁、特许经营、资产证券化等融资模式。发挥南宁产业发展基金、城市发展基金等基金作用，加快推动华润基金等实质性运作。大力培育和发展资本市场，利用好资本市场扶贫绿色通道政策，推动更多优质企业上市挂牌，推进上市挂牌企业再融资和并购重组；支持符合条件的企业利用债券市场、区域性股权市场、私募市场扩大直接融资规模。持续推进“4321”政府性融资担保体系建设，发挥“两台一会”平台优势，推动南宁市中小微企业孵化基金募资和投放，拓宽专利权质押融资渠道，努力解决中小微企业融资难融资贵问题。

　　着力优化营商环境。提升政策环境，努力破除歧视性限制和各种隐性障碍，大力支持民营企业发展，构建“亲”“清”新型政商关系。优化政务环境，持续推进“放管服”改革，加快明确行政许可权的审批与监管职责边界，推进行政审批和监管信息共享，建立健全审管联动制度；持续推进行政审批流程优化再造，强化政府部门权责清单“两单融合”后续管理，全面实施“双随机一公开”监管机制；深化商事制度改革，推动国家级开发区开展“证照分离”改革试点。改善经营环境，加快建成统一规范的市县公共资源电子交易平台；加快信用体系建设，建立健全守信联合激励和失信联合惩戒制度，加快推进在行政管理中使用信用记录和信用报告；实施质量强市战略和商标品牌战略，培育一批具有市场竞争力的品牌产品。

　　（四）推动形成全面开放新格局，持续推进“南宁渠道”升级。

　　按照自治区“四维支撑、四沿联动”布局，大力实施全方位、宽领域、多层次的开放发展战略，加快打造“南宁渠道”升级版。

　　增强平台支撑作用。建好用好开放平台，服务好第15届中国—东盟博览会和商务与投资峰会，力争五象新区申报国家级新区成功，落实经开区申报临空经济示范区和核准区扩区等工作，积极申报中国（南宁）跨境电子商务综合试验区。加快中国—东盟信息港南宁核心基地建设，以信息港小镇和大数据中心建设为抓手，建设数字经济基地，着力推进中国—东盟检验检测认证高技术服务集聚区、中国电信东盟国际信息园、新型智慧城市协同创新中心等重点项目建设。加快完善南宁综合保税区配套设施，引进一批规模型电商企业和加工贸易企业。推进吴圩空港经济区重点产业发展区域建设，加快产业集聚。

　　拓展区域合作空间。积极参与中新互联互通“南向通道”建设。加强与西南中南地区在交通物流、文化旅游等领域的产业合作。落实“双核驱动”战略，持续深化北部湾经济区综合配套改革；深度参与珠江—西江经济带建设，落实粤桂共同行动计划。加强与北部湾城市群的协同发展，深化与粤港澳大湾区的融合发展，加快研究建立邕广深合作直通平台。加强与港澳台经贸往来，积极培育自治区级CEPA先行先试示范基地。

　　大力发展开放型经济。加快建设内陆开放型经济战略高地，深入实施第二轮加工贸易倍增计划，主动承接珠三角地区产业转移，大力发展软件和信息技术、金融、文化创意设计等领域服务外包，打造外贸发展新引擎。支持高新技术产品和机电产品出口，提升出口质量和附加值。推进广西—东盟区域（南宁）外贸一体化综合体通关提速工程上线运行，促进贸易便利化。坚持“引进来”与“走出去”并重，深入实施招商引资三年行动计划，围绕重点产业和重点领域，突出建链、补链、延链、强链，加强产业链招商和以商招商，推动市县工业园区联动招商，加快建设南宁市投资信息服务中心；鼓励优质企业积极参与国际产能合作，支持铝业、机械、食品等优势产业资本到境外投资建厂。

　　（五）坚持生态优先绿色发展，持续推进绿城品质升级。

　　深入践行绿水青山就是金山银山的理念，坚持“治水、建城、为民”的城市工作主线，积极创建国家生态文明建设示范市，建设美丽南宁。

　　确保五象新区核心区基本成型。进一步推进新区产城融合，着力推动新区从量的扩张向质的提升转变、从抓具体项目建设向统筹全面可持续发展转变。总部基地金融街基本成型，高层建筑主体结构封顶率达75%以上，一批企业入驻办公、一批商业项目开业，五象商圈初显。加快建设广西—东盟地理信息与卫星应用产业园、南宁启迪东盟科技城等一批产业园，加快推进宜家家居、招商物流等项目，加快集聚先进装备制造、新材料、新能源等新兴产业。加快完善新区基础设施、公共服务配套，续建广西医科大学东盟国际口腔医学院，建成总部基地地下空间、第四实验小学、邻里中心等一批项目，给排水、燃气、电力、公交场站、农贸市场、垃圾转运站、公厕等配套设施同步建成。继续推进国家级绿色生态示范区创建，初步呈现现代、生态、便利、特色的宜居宜业的城市新区形象。

　　全力以赴办好第十二届中国（南宁）国际园林博览会。紧扣“生态宜居 园林圆梦”主题，紧盯8月园区试运营、12月全面建成开园的目标，全力打造“生态的园博、文化的园博、共享的园博”。重点加快展园区、遗址公园区、田园风光区等片区的主体场馆、园林绿化建设，加大力度整治园区周边市容环境，确保周边路网及快速公交2号线等配套工程如期建成使用。统筹做好园博园建设、园博会筹办及展后的持续运营管理工作，办成永不落幕的园博会。

　　坚决打好污染防治攻坚战。打赢蓝天保卫战。抓好大气污染联防联控，健全扬尘污染治理长效机制，注重“智慧治尘”，推进市区网格化监测，加大对工地到消纳场“两点一线”、工业及交通排放等污染的整治力度，确保空气质量优良率91%以上，PM10、PM2.5年均浓度分别不超过61微克/立方米、35微克/立方米。提升绿城品质。承办好2018年中国生态文明论坛（南宁）年会。深化国家森林城市和国家生态园林城市建设，全面提升城市主干道路、街区和公园的景观效果，实施重要道路及节点四季鲜花种植项目。持续加大黄土裸露整治及超高土治理力度，继续推进立体绿化建设，提升城市绿化品位和档次。加快湿地保护规划实施，推进大王滩国家湿地公园试点建设。打造提升五象岭森林公园，继续推进青秀山风景区建设，强化对大明山等自然保护区的规范化管理，构建城乡一体化生态格局。做好水的文章。全面完成邕江两岸148公里岸线综合整治，打造“百里秀美邕江”景观带，建成亭子旅游码头，打造邕江水上观光游线路。巩固扩大海绵城市建设试点成果，确保顺利通过国家验收。整治内河两岸乱象，巩固建成区黑臭水体治理成效，启动建成区外18.8公里黑臭水体治理。建设完善河长制信息平台，按照一河（江、湖、库）一策要求，全面落实河长制、湖长制各项工作。完成邕江取水口上移工程，确保城市饮用水安全。积极营建水源涵养林，降低饮用水水源保护区范围内的森林经营强度，保持集中式饮用水水源水质达到或优于Ⅲ类比例达100%。深化生态环境综合治理。深入实施“水十条”，全面实施“土十条”，严格落实生态保护红线、永久基本农田、城镇开发边界三条控制线，贯彻实施环境保护税法，坚决制止和惩处破坏生态环境行为。实施山水林田湖草生态保护与修复工程。继续推进土地整治、表土剥离、土地复垦等工作。开展建筑施工噪声污染专项整治等行动，减少噪声污染。持续深化美丽乡村建设，实施化肥农药使用量零增长行动，加强农业面源污染治理和土壤重金属污染防治，综合治理乡村环境。完成生态环境监管体制改革，落实生态环境保护目标管理责任制。巩固中央环保督察反馈意见整改成效，切实防止问题反弹。

　　提升城市综合承载能力。坚持规划引领。围绕建设北部湾城市群核心城市，高标准推进中心城控规等各项规划编制工作，力争年底前将新一轮城市总规成果上报国务院审批。优化建设用地布局，精准有效开展存量土地和城镇低效用地盘活工作，建立用地保障共同责任机制。让城市更通畅便捷。加快完善城市路网，完成高速公路改快速路、快环综合整治项目重要节点改造等项目建设，加快主干道建设及旧城区道路改造扩建。落实公交优先发展战略，量力而行、稳步推进轨道交通2号线东延长线和3、4、5号线一期工程建设，开展轨道交通机场专线、教育园区专线项目前期工作，优化公交站点和线网布局，建设立体综合交通枢纽，提升公共交通分担率，不断完善城市绿道等城市慢行交通设施。持续推进城市更新。加快中山路片区等历史文化街区保护改造，稳步推进棚户区改造、旧城改造三年计划。加快城市燃气、油气等管网改造提升工作，创新推进地下综合管廊的建设运营，12个地下综合管廊试点项目全部投入使用。继续推进以BIM等信息技术为支撑的工程质量提升工作。加快城市治理现代化。深化市县城市管理和执法体制、生态环境监管体制改革，推进综合执法。健全政府主导、第三方参与的城建计划精细化管理系统，努力解决市政重复投资和“马路拉链”问题。大力推进新型智慧城市建设，提升医疗、教育、社保、交通、城管、气象等领域的智慧化运用。推进市政污水管网雨污分流改造，加快江南、埌东、三塘等污水处理厂扩容提标改造和“十三五”第二批镇级污水处理设施建设。推进生活垃圾强制分类。扎实推进“厕所革命”。加大“两违”巡查监控和整治力度，深化“美丽南宁·整洁畅通有序大行动”。

　　（六）实施乡村振兴战略，发展壮大县域经济。

　　坚持农业农村优先发展，按照“产业兴旺、生态宜居、乡风文明、治理有效、生活富裕”总要求，推动城乡融合发展，加快推进农业农村现代化。

　　构建新型农业生产经营体系。科学制定乡村振兴战略规划。深化农村土地制度改革，落实农村承包地“三权”分置制度，保持土地承包关系稳定并长久不变。深化农村集体产权制度改革，推进农村不动产登记工作，有序推进农村土地流转，保障农民财产权益。稳步推进集体林权制度改革，做好宾阳县集体林地“三权”分置试点工作。巩固国有林场改革成果，确保通过国家和自治区验收。扶持培育新型农业经营主体，积极探索农业产业化联合体，力争新增认定市级以上农业产业化龙头企业10家、农民合作社400家、家庭农场150家。加快培育新型职业农民，打造一支懂农业、爱农村、爱农民的“三农”工作队伍。

　　强化县域产业支撑。大力实施县域经济三年行动计划。突出县域产业集聚发展，加强县域工业园区规划建设，支持六景、黎塘、伶俐工业园区等县域工业园区做大做强，引导产业向园区集聚。推动县域产业开放发展，引导各县主动承接发达地区产业转移，实施农产品加工、园区支柱产业培育、全域旅游发展、健康产业发展、生态产业发展五大工程，打造“一县一拳头产业”。加强县域与中心城区的产业联动，推进各县围绕市级重点产业大力发展相关配套产业，各培育形成一条以上优势产业链，推动我市县域经济在全区争先进位。

　　加快县域新型城镇化。深入实施主体功能区规划，健全城乡融合发展体制机制，大力推进城乡建设用地增减挂钩，清除阻碍要素下乡各种障碍。完善城镇体系架构，推进县域扩容提质，打造一批支撑县域经济发展的产业重镇、文化旅游名镇。引导特色小镇健康发展，加快横县六景镇第三批国家新型城镇化试点和校椅镇国家特色小镇建设，继续打造宾阳县新型城镇化示范县，完成武鸣区罗波镇、宾阳县古辣镇等自治区百镇建设示范工程，推进马山县古零镇等市级特色小镇培育建设。夯实县域基础设施支撑，落实自治区县域基础设施和公共服务设施提升三年行动计划项目，扎实推进“四好农村路”建设，加快完善能源、农田水利、信息网络等基础设施，建立和完善基础设施后续管理工作机制。加快户籍制度改革落地步伐，推进农业转移人口市民化进程。谋划启动幸福乡村活动，打造美丽南宁乡村建设升级版。

　　（七）坚持问题导向，持续推进深化改革升级。

　　坚定改革方向，科学统筹各项改革任务，力争在重点领域和关键环节取得突破性进展，不断增强经济社会发展动力活力。

　　推动“三去一降一补”取得新成效。出台并实施市本级供给侧结构性改革“1+5”政策文件。强化行业准入管理，确保重点去产能行业无违规新增产能，整合木薯淀粉酒精等过剩产能。推动“三供一业”分离移交，全面完成99家国有“僵尸企业”低效无效产能出清，建立非国有“僵尸企业”处置机制。加大力度化解非住宅商品房库存。做好降本减负各项工作，切实减轻企业负担。加快补齐公共服务、基础设施等方面短板。

　　持续加强重点领域改革。全面深化国资国企改革。改革国有资本授权经营体制，推进国有资本经营公司试点和混合所有制试点。推动国有资本做强做优做大。深化公共资产负债管理智能云平台建设，不断拓展内涵和外延，推进财政、国资、扶贫资金管理等系统一体化建设，确保公共资产“摸得清、来去明、管得住、利用好”。加快完善以管资本为主的国资监管体制，加强事中、事后监管。突出抓好财政改革。承接做好中央、自治区在具体领域的财政事权和支出责任划分改革，稳步推进市以下财政事权和支出责任划分改革。深化预算绩效管理改革，继续扩大试点覆盖面。加快政府购买服务改革，支持事业单位和社会组织承接政府购买服务。加快推进金融改革。建设沿边金融综合改革试验区升级版，扩大中国-东盟（南宁）货币指数应用，加快建设中国—东盟（南宁）金融服务平台。支持金融机构为“一带一路”沿线国家城市和企业提供跨境金融服务。争取开展绿色金融试点，创新发展绿色金融产品，推动绿色企业上市。引导传统金融机构依托互联网转型升级，规范发展互联网金融。深化不动产登记改革。创新“互联网+不动产登记”模式，构建“一点为中心、多点辐射”的布设格局。

　　打好防范化解重大风险攻坚战。加强地方金融监管能力建设，创新和完善金融监管机制，做好金融重点领域风险防范和处置，加大预防、打击和处置非法集资力度，有效打击违法违规金融活动和恶意逃废债务行为，牢牢守住不发生系统性金融风险的底线。加强政府性债务风险评估、预警和监管。坚持房子是用来住的、不是用来炒的定位，保持房地产市场调控政策的连续性和稳定性，积极培育和发展住房租赁市场，合理引导市场预期和购房行为，全面推行存量房交易网签管理，促进房地产市场平稳健康发展。

　　（八）坚持精准施策，持续推进民生福祉升级。

　　坚持以人民为中心的发展思想，多谋民生之利、多解民生之忧。针对人民群众关心的热点难点问题精准施策，不断提升群众的获得感幸福感安全感。

　　坚决打好精准脱贫攻坚战。持续推进“七个一批”“七大工程”，扎实推进年度脱贫摘帽滚动计划实施，加大扶贫资金投入，推动扶贫同扶志、扶智相结合。全力推进易地扶贫搬迁，盯紧盯牢进度、质量、资金三个关键，强化“八包”责任，提高建设竣工率和搬迁入住率，加强搬迁人口后续产业、就业扶持，确保贫困群众搬得出、稳得住、有事做、能致富。集中攻克深度贫困地区脱贫任务，将新增脱贫攻坚资金、项目、举措向深度贫困地区集中，重点落实好企业与隆安县都结乡以及56个深度贫困村的结对帮扶工作，确保深度贫困村脱贫摘帽不掉队。扎实推进产业扶贫，推动每个贫困村发展1-3个特色产业，力争做到有劳动能力的贫困户家家有稳定生产经营收入。加快发展贫困村村级集体经济，确保年度脱贫的贫困村村级集体经济收入达到摘帽标准。推进粤桂扶贫协作，健全茂名—南宁“携手奔小康”对口结对帮扶工作机制，实施“贫困村创业致富带头人”试点工程。全面完成自治区下达的年度脱贫攻坚任务。

　　优先发展教育事业。实施第三期学前教育行动计划，持续扩大多元普惠幼儿园覆盖面。深入推进义务教育学区制管理改革，加快推进“全面改薄”工程，进一步推进义务教育均衡发展，力争5个县区通过义务教育均衡发展国家督导评估认定。实施高中阶段教育提质发展计划，推动普通高中多样性、特色化、优质化发展。着力解决“择校热”“大班额”“大通铺”等突出问题。加快发展现代职业教育，推进中高职教育与重点产业深度融合发展，继续支持南宁学院创建南宁大学。持续实施教育管办评分离等重大教育改革项目。促进南宁教育园区新开工5所学校，加快建设广西医科大学武鸣校区等一批学校。

　　加快建设健康南宁。健全重大疾病防控机制，重点做好艾滋病、结核病、手足口病等全市重点传染病防控与疫情处置。完善分级诊疗制度，加快组建市级医疗集团、县域医疗共同体。加强全科医生队伍建设，提高基层医疗机构服务能力。加快医保支付方式改革，推行按病种付费为主的多元复合式医保支付方式。全面推进智慧健康信息工程，加快医疗信息共建互享。保障重点人群健康，着力解决婴幼儿照护和儿童早期教育服务问题，广泛开展残疾人康复服务。发展以医养结合为重点的健康养老模式，推进公办示范性养老机构建设，加快建设五象新区、东盟经开区养老产业集聚区，打造一批养老服务业龙头企业和特色品牌。大力发展中医药及健康产业，扶持中西医、壮瑶医事业发展。积极开展爱国卫生运动，全力争创国家卫生城市“三连冠”。大力发展体育事业，深入实施全民健身计划，推进全民健身和全民健康深度融合试点城市建设。抓好体育产业发展，着力打造赛事品牌，办好环广西公路自行车世界巡回赛（南宁站）、2018年“中国杯”国际足球锦标赛、南宁国际马拉松赛等一批重大国际赛事。

　　大力促进创业就业。积极创建全民创业城市和充分就业城市，推进“邕城创业行”等品牌建设，重点扶持高校毕业生、返乡农民工等群体创业。全面落实“岗位拓展计划”“创业引领计划”和“就业服务与援助计划”，着力解决结构性就业矛盾，实现更高质量和更充分就业。

　　健全社会保障体系。持续推进全民参保计划。加快完善医保政策，扩大异地就医住院医疗费用直接结算的定点医疗机构范围和职工大额医疗费用统筹支付范围。健全完善社会救助体系，提高农村低保保障标准和城乡低保补助水平。落实好医疗救助制度和特困人员救助供养制度。全力抓好保障性安居工程建设、分配入住和运营管理等工作。加快市第二福利院等项目建设。

　　提高文化软实力。坚持中国特色社会主义文化发展道路，坚定文化自信，培育和践行社会主义核心价值观。大力发展文化事业，加快完善公共文化服务体系，加强全民阅读的推广和引导，继续办好民歌湖大舞台周周演等群众性文化活动，打造《朱槿花开》《刘三姐》等一批文艺精品。积极发展文化产业，充分发挥广西文化艺术中心的作用，不断满足市民对高品质文化的向往和需求。积极推进文化对外交流，加快建设中国—东盟文化交流协作机制平台，筹办好第20届南宁国际民歌艺术节等活动。弘扬和挖掘优秀传统文化，着力做好“顶蛳山”“壮族歌圩”等地方特色文化的传承保护，推进国家历史文化名城申报工作。

　　推进社会治理创新。深入推进平安南宁建设，加快建设“智慧警务”，健全完善“七位一体”立体化社会治安防控体系，坚决从严打击黑恶势力、严重暴力犯罪及黄赌毒、网上虚假信息诈骗、倒卖个人信息、传销等违法犯罪行为。加大矛盾纠纷化解和调处力度，进一步畅通和规范群众诉求表达渠道。推进防灾减灾救灾管理体制机制改革，全面提升综合防灾减灾救灾能力。全面提升国防动员能力，抓好优抚安置政策落实，推动军民深度融合发展。健全安全风险分级管控和隐患排查治理双重预防性工作机制，坚决防范重特大安全事故发生。全面实施食品安全战略，积极推动食品安全追溯体系建设，加快创建国家、自治区食品安全示范城市。继续推进“诚信经营、放心消费”创建活动，规范和维护市场经济秩序。巩固创建民族团结进步示范市成果，依法管理宗教事务，促进民族团结和谐稳定。

　　继续做好为民办实事工程。坚持财政支出更多向民生倾斜，全力推进教育、健康等10大项为民办实事工程，让发展成果更多惠及百姓。

　　三、建设服务型政府

　　打铁必须自身硬。我们将以党的十九大精神为统领，坚持依法行政，转变政府职能，增强政府公信力和执行力，加快建设人民满意的服务型政府。

　　旗帜鲜明讲政治。把坚决维护以习近平同志为核心的党中央权威和集中统一领导作为最大的政治、最重要的大局，引导政府系统广大党员干部自觉增强“四个意识”，坚定“四个自信”，自觉做习近平新时代中国特色社会主义思想的坚定信仰者和忠实实践者，不折不扣贯彻落实党中央决策部署。深入学习贯彻党的十九大精神，在学懂弄通做实上下功夫，紧扣我市发展方向和工作重心，深入谋划新时代我市发展新举措，让党的十九大精神在南宁落地生根、开花结果。

　　全面推进法治政府建设。深入实施法治政府建设工作方案，持续推进法治南宁升级。加快完善政府立法工作机制，加强城乡建设与管理、环保等重点领域精细化立法，继续拓展社会各方有序参与政府立法的途径和方式。完善公共法律服务体系，落实“谁执法谁普法”责任制，深入实施“七五”普法。严格履行重大行政决策程序规定。不断规范行政执法行为。自觉接受市人大及其常委会法律监督和工作监督，主动接受人民政协民主监督和社会舆论监督，切实做好人大代表议案建议和政协提案办理等工作。加强政府参事、专家咨询委员会等新型智库建设，发挥好政府法律顾问作用，提高依法科学决策水平。

　　提升政务服务水平。推动政务服务体系标准化建设，着力提供无差异、均等化政务服务。开展清理规范证明事项工作，清理取消教育、就业、社保、居住等领域的各种不必要证明。加快建设“互联网+政务服务”，推进信息系统整合和政务数据共享，逐步推动网上政务服务平台向基层延伸，推动更多涉企涉民政务服务上线。提升南宁市民中心软硬件水平，优化“一窗受理、并联审批、限时办结、一窗出件”的一站式服务。深化政务公开，加强新闻发布，加强重大涉民政策的解读与宣传，推动政务决策、执行、管理、服务和结果“五公开”。

　　持之以恒改进作风。严格落实党风廉政建设主体责任，切实履行“一岗双责”，加强和规范党内政治生活。巩固和拓展落实中央八项规定精神成果，驰而不息纠正“四风”，认真查找“四风”突出问题特别是形式主义、官僚主义新表现，扎实开展扶贫领域腐败和作风问题专项治理。加强对重点领域、关键岗位的审计和监督执纪问责。结合推进“两学一做”学习教育常态化制度化，开展“不忘初心、牢记使命”主题教育，拓宽培训渠道、创新教育形式，着力提升干部队伍履职尽责能力。落实容错纠错机制，健全激励机制，大力营造想干事、敢干事、干成事的良好氛围。

　　各位代表！新时代要有新气象，更要展现新作为。让我们更加紧密团结在以习近平同志为核心的党中央周围，在中央、自治区和市委的坚强领导下，不忘初心、牢记使命，攻坚克难、奋力拼搏，以新担当开启新征程、以新作为推动新发展，持续推进“六大升级”工程，加快建设“四个城市”，勇当广西持续营造“三大生态”、加快实现“两个建成”的排头兵，奋力推动首府经济社会向更高质量发展，以优异成绩迎接改革开放40周年和自治区成立60周年！
